# Trends in CD4 and viral load testing 2005 to 2018: multi‐cohort study of people living with HIV in Southern Africa

**DOI:** 10.1002/jia2.25546

**Published:** 2020-07-08

**Authors:** Elizabeth Zaniewski, Cam H Dao Ostinelli, Frédérique Chammartin, Nicola Maxwell, Mary‐Ann Davies, Jonathan Euvrard, Janneke van Dijk, Samuel Bosomprah, Sam Phiri, Frank Tanser, Nosisa Sipambo, Josephine Muhairwe, Geoffrey Fatti, Hans Prozesky, Robin Wood, Nathan Ford, Matthew P Fox, Matthias Egger

**Affiliations:** ^1^ Institute of Social and Preventive Medicine University of Bern Bern Switzerland; ^2^ Centre for Infectious Disease Epidemiology and Research School of Public Health and Family Medicine University of Cape Town Cape Town South Africa; ^3^ SolidarMed Masvingo Zimbabwe; ^4^ Centre for Infectious Disease Research in Zambia Lusaka Zambia; ^5^ Department of Biostatistics School of Public Health University of Ghana Accra Ghana; ^6^ Lighthouse Lilongwe Malawi; ^7^ Africa Health Research Institute KwaZulu‐Natal South Africa; ^8^ Lincoln International Institute for Rural Health University of Lincoln Lincoln United Kingdom; ^9^ School of Nursing and Public Health University of KwaZulu‐Natal Durban South Africa; ^10^ Centre for the AIDS Programme of Research in South Africa (CAPRISA) University of KwaZulu‐Natal Durban South Africa; ^11^ Chris Hani Baragwanath Academic Hospital Johannesburg South Africa; ^12^ SolidarMed Maseru Lesotho; ^13^ Kheth’Impilo AIDS Free Living Cape Town South Africa; ^14^ Division of Epidemiology and Biostatistics Department of Global Health Faculty of Medicine and Health Sciences Stellenbosch University Cape Town South Africa; ^15^ Division of Infectious Diseases Department of Medicine Stellenbosch University Cape Town South Africa; ^16^ Gugulethu ART Programme (Desmond Tutu HIV Centre) Cape Town South Africa; ^17^ Department of HIV/AIDS and Global Hepatitis Programme World Health Organization Geneva Switzerland; ^18^ Department of Global Health Boston University Boston MA USA; ^19^ Department of Epidemiology Boston University Boston MA USA; ^20^ Health Economics and Epidemiology Research Office Department of Internal Medicine School of Clinical Medicine Faculty of Health Sciences University of the Witwatersrand Johannesburg South Africa

**Keywords:** CD4 lymphocyte count, viral load, Africa, Southern, antiretroviral therapy, highly active, Cohort studies, HIV infections

## Abstract

**Introduction:**

The World Health Organization (WHO) recommends a CD4 cell count before starting antiretroviral therapy (ART) to detect advanced HIV disease, and routine viral load (VL) testing following ART initiation to detect treatment failure. Donor support for CD4 testing has declined to prioritize access to VL monitoring. We examined trends in CD4 and VL testing among adults (≥15 years of age) starting ART in Southern Africa.

**Methods:**

We analysed data from 14 HIV treatment programmes in Lesotho, Malawi, Mozambique, South Africa, Zambia and Zimbabwe in 2005 to 2018. We examined the frequency of CD4 and VL testing, the percentage of adults with CD4 or VL tests, and among those having a test, the percentage starting ART with advanced HIV disease (CD4 count <200 cells/mm^3^) or failing to suppress viral replication (>1000 HIV‐RNA copies/mL) after ART initiation. We used mixed effect logistic regression to assess time trends adjusted for age and sex.

**Results:**

Among 502,456 adults, the percentage with CD4 testing at ART initiation decreased from a high of 78.1% in 2008 to a low of 38.0% in 2017; the probability declined by 14% each year (odds ratio (OR) 0.86; 95% CI 0.86 to 0.86). Frequency of CD4 testing also declined. The percentage starting ART with advanced HIV disease declined from 83.3% in 2005 to 23.5% in 2018; each year the probability declined by 20% (OR 0.80; 95% CI 0.80 to 0.81). VL testing after starting ART varied; 61.0% of adults in South Africa and 10.7% in Malawi were tested, but fewer than 2% were tested in the other four countries. The probability of VL testing after ART start increased only modestly each year (OR 1.06; 95% CI 1.05 to 1.06). The percentage with unsuppressed VL was 8.6%. There was no evidence of a decrease in unsuppressed VL over time (OR 1.00; 95% CI 0.99 to 1.01).

**Conclusions:**

CD4 cell counting declined over time, including testing at the start of ART, despite the fact that many patients still initiated ART with advanced HIV disease. Without CD4 testing and expanded VL testing many patients with advanced HIV disease and treatment failure may go undetected, threatening the effectiveness of ART in sub‐Saharan Africa.

## 1 INTRODUCTION

The World Health Organization (WHO) has recommended immediate initiation of antiretroviral therapy (ART) for all people living with HIV since 2015, regardless of CD4 cell count or clinical stage [1], but WHO still recommends CD4 cell counts at enrolment into HIV care. The CD4 cell count is the best way to measure a patient’s immune status prior to starting ART or return to care following treatment interruption, and is the best predictor of disease progression and risk of death, especially among those with advanced HIV disease [2, 3, 4]. CD4 cell count is used to guide a number of prophylactic and diagnostic interventions, including prioritizing testing for opportunistic infections, such as Cryptococcus or tuberculosis [5, 6].

The primary benefit of ART is the suppression of HIV‐1 viral replication [1, 7, 8]. Suppression of viral load (VL) (defined by WHO as ≤1000 HIV‐RNA copies/mL) reduces morbidity and mortality among patients living with HIV and onward transmission of the virus [9]. It is the third of the UNAIDS global 90‐90‐90 and 95‐95‐95 targets launched in 2014, which aim to have 73% and 86%, of all people living with HIV virally suppressed by 2020 and 2030 respectively [10, 11]. Since 2013, WHO has recommended VL testing, rather than CD4 testing, as the preferred monitoring approach to detect treatment failure following ART initiation [12]. WHO guidelines were further refined in 2016 and currently recommend routine VL testing six months after starting ART and yearly thereafter among patients stable on ART [1, 7]. Patients with two consecutive unsuppressed VL measurements (>1000 copies/mL) despite enhanced adherence counselling, taken within a three‐month interval and at least six months after starting ART are considered to be in treatment failure [1, 7, 12]. Treatment failure indicates either some degree of drug resistance, or that ART has not been taken properly [13].

The President’s Emergency Plan for AIDS Relief (PEPFAR) [14] has been one of the largest financial commitments to combat HIV/AIDS, supporting more than 50 countries globally, including more than 20 countries in Africa [14, 15]. In 2018, PEPFAR shifted testing priorities reducing the overall level of support for CD4 testing to prioritize access to VL monitoring [16, 17]. In this study, we examined longitudinal data from six countries in the International epidemiology Databases to Evaluate AIDS (IeDEA) Southern Africa region, all supported by PEPFAR since 2008, to assess trends in CD4 and VL testing between 2005 and 2018.

## 2 METHODS

### 2.1 Data sources

The IeDEA collaboration (https://www.iedea.org/) is an international research and data consortium funded by the National Institutes of Health since 2006 [18, 19, 20]. IeDEA is comprised of seven regional data centres that collect, pool and analyse existing clinical and epidemiological data on people living with HIV under care in routine settings as a cost‐effective way of generating large electronic data sets to address high priority research questions in HIV/AIDS treatment and care that cannot be answered by single cohorts [21]. Patient‐level data, including patient characteristics, clinic visit history, laboratory test measurements and medications are collected from primary through tertiary HIV care facilities across both urban and rural settings [21]. The IeDEA Southern Africa region currently contains patient‐level data on more than one million adults and children living with HIV from 17 large treatment programmes across Lesotho, Malawi, Mozambique, South Africa, Zambia and Zimbabwe [22].

### 2.2 Eligibility criteria

We included all CD4 cell count and VL test measurements from adults aged ≥15 years who were ART‐naïve at enrolment and started ART between 2005 and 2018 at one of the HIV treatment programmes currently supported by IeDEA Southern Africa. We only included public‐access programmes that collect adult CD4 and VL test data using government (i.e. not privately funded) laboratory services. To ensure adequate time to capture test measurements we excluded adults who started ART less than one week before the date of database closure and adults who had less than six months of possible test measurement data available prior to ART start (e.g. patients who started ART in the first six months of 2005).

### 2.3 Outcomes

We assessed the number of CD4 cell count and VL tests performed by exact calendar date from 1 January 2005 to 31 December 2018, irrespective of whether testing occurred before or after ART start, and calculated the ratio of CD4 cell count to VL testing per year over time and stratified by country. We examined the percentage of adults with a CD4 cell count at ART start, the percentage of adults with any CD4 cell count up to six months before ART start, and, among those with a CD4 cell count, the percentage of adults starting ART with advance HIV disease by country and calendar year. We defined ART as a regimen of at least three antiretroviral drugs from two drug classes, and advanced HIV disease as a CD4 cell count <200 cells/mm^3^.

We defined CD4 cell count at ART start as a CD4 cell count taken within a time window of three months before and up to one week after ART start. In earlier years, it may have taken several months to initiate ART because of eligibility requirements and extensive preparation prior to initiation of therapy [23]. To ensure we captured these earlier CD4 cell count measurements, we widened the time window to six months before ART start to assess the percentage of adults with any CD4 cell count before ART start. If an adult had multiple CD4 cell count measurements within a time window, we selected the CD4 cell count measurement closest to ART start.

WHO recommends VL testing six months after ART start; however in some parts of Southern Africa regional guidelines recommend the first VL test at three months for pregnant and breastfeeding women, and at four months for adults aged ≥15 years [24]. We therefore assessed the percentage of adults with a VL test three to nine months after ART initiation to ensure we captured all recommended testing periods and to allow up to a three‐month delay in testing. Among those with a VL test, we calculated the percentage with an unsuppressed VL (>1000 HIV‐1 RNA copies/mL). To allow sufficient follow‐up time to undertake VL testing, we excluded adults who started ART less than nine months before database closure from the analysis of VL testing. We also excluded adults who started ART in 2018 because of the small sample size; the analysis of VL testing thus covered adults starting ART up to 31 December 2017 and VL testing data up to nine months thereafter. If multiple VL measurements occurred within the time window, we selected the first eligible VL measurement.

### 2.4 Statistical methods

We used descriptive statistics to summarize baseline characteristics at ART initiation, including percentage female, median age, median follow‐up time and year of ART start stratified by country. We also used descriptive statistics to characterize trends in CD4 and VL testing frequency and the proportion of adults with each test stratified by country. We used a mixed effect logistic regression model with random intercepts by country to assess the probability of having a CD4 cell count at ART start, a VL test after ART start, and among those with a test, the probability of having advanced HIV disease or unsuppressed VL over calendar year adjusted for age and sex. We created three age categories: <25, 25 to 49 and >49 years of age. We defined sex as either male or female and excluded 111 patients with unknown sex or date of birth. All statistical analyses were performed using Stata version 15.1 (Stata Corp., College Station, TX, USA).

### 2.5 Ethical considerations

Local review boards and ethics committees approved the use of the data included in this study. The Cantonal Ethics Committee of the Canton of Bern, Switzerland, approved data merging and the collaborative analyses. Local review boards and the Cantonal Ethics Committee of the Canton of Bern waived the requirement to obtain informed consent.

## 3 RESULTS

Our main analysis included 502,456 adults from 14 programmes encompassing more than 300 clinics across six countries in Southern Africa (Table 1). Three programmes were either paediatric or private and thus excluded from analyses. Median (IQR) age at ART initiation was 34.3 (28.6 to 41.3) years and 63.7% of patients were female. Median (IQR) follow‐up time was 59.1 (28.8 to 95.3) months, ranging from 39.6 (20.6 to 60.7) months in Mozambique to 74.5 (39.3 to 107.8) months in South Africa. Most adults initiated ART in years 2009 to 2013 (43.2%), followed by years 2014 to 2018 (38.4%), and 2005 to 2008 (18.4%). The analysis of VL testing after ART start included 458,528 adults (91.3% of adults included in the CD4 count analysis). Patient characteristics were similar but, as expected as a result of the broader exclusion criteria, the median follow‐up time was longer (65.1 months) than in the CD4 count analysis (59.1 months) (Table S1).

**Table 1:** Characteristics of adult patients at antiretroviral therapy (ART) initiation by country 2005 to 2018

	Lesotho	Malawi	Mozambique	South Africa	Zambia	Zimbabwe	Total
Total No. of patients	12,441 (100%)	80,365 (100%)	16,178 (100%)	115,667 (100%)	251,816 (100%)	25,989 (100%)	502,456 (100%)
Female	8180 (65.8%)	49,137 (61.1%)	10,934 (67.6%)	77,763 (67.2%)	156,597 (62.2%)	17,689 (68.1%)	320,300 (63.7%)
Age group in years							
<25	1101 (8.9%)	10,960 (13.6%)	3812 (23.6%)	12,753 (11.0%)	31,141 (12.4%)	2938 (11.3%)	62,705 (12.5%)
25 to 49	8686 (69.8%)	61,683 (76.8%)	10,915 (67.4%)	92,500 (80.0%)	199,950 (79.4%)	18,931 (72.8%)	392,665 (78.1%)
>49	2654 (21.3%)	7722 (9.6%)	1451 (9.0%)	10,414 (9.0%)	20,725 (8.2%)	4120 (15.9%)	47,086 (9.4%)
Median (IQR) age in years	37.8 (30.7 to 47.8)	34.0 (28.3 to 41.0)	30.9 (25.2 to 39.6)	34.2 (28.6 to 41.3)	34.2 (28.7 to 40.9)	36.9 (30.2 to 44.7)	34.3 (28.6 to 41.3)
Period of ART initiation							
2005 to 2008	1880 (15.1%)	8191 (10.2%)	1075 (6.7%)	27,481 (23.8%)	52,328 (20.8%)	1607 (6.2%)	92,562 (18.4%)
2009 to 2013	4990 (40.1%)	37,411 (46.5%)	4227 (26.1%)	56,237 (48.6%)	101,864 (40.4%)	12,464 (48.0%)	217,193 (43.2%)
2014 to 2018	5571 (44.8%)	34,763 (43.3%)	10,876 (67.2%)	31,949 (27.6%)	97,624 (38.8%)	11,918 (45.8%)	192,701 (38.4%)
Median (IQR) follow‐up in months	40.6 (9.2 to 77.1)	48.9 (21.1 to 78.1)	39.6 (20.6 to 60.7)	74.5 (39.3 to 107.8)	59.6 (28.8 to 98.4)	57.8 (31.9 to 83.7)	59.1 (28.8 to 95.3)

Number of patients (%) are shown unless otherwise indicated.

### 3.1 Frequency of CD4 cell count and VL testing

**Figure 1:**
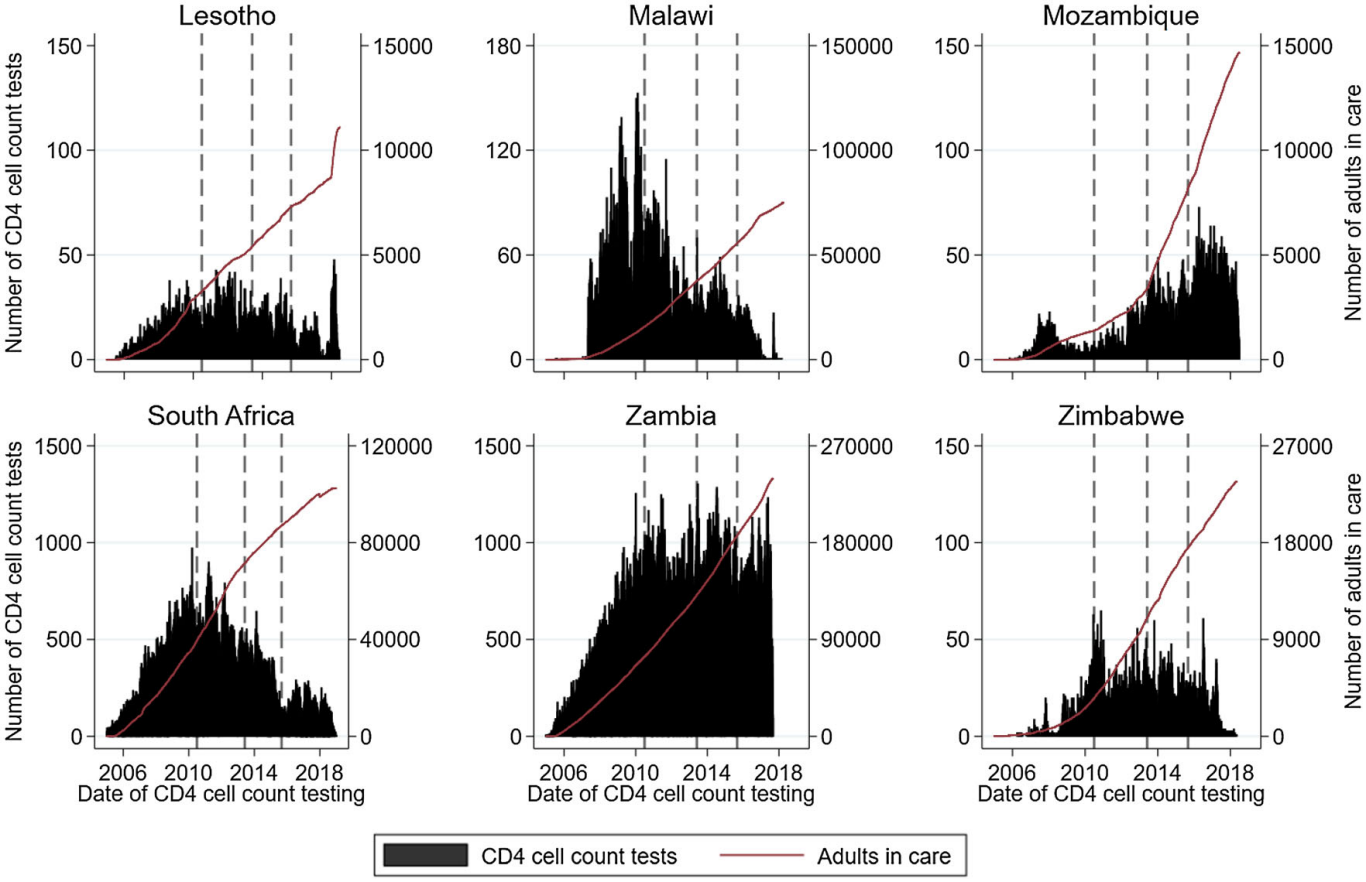
Frequency of CD4 cell count testing per day and cumulative number of adult patients in care by country. The vertical lines indicate the change in WHO guidelines. Scale of y‐axis differs across countries.

**Figure 2:**
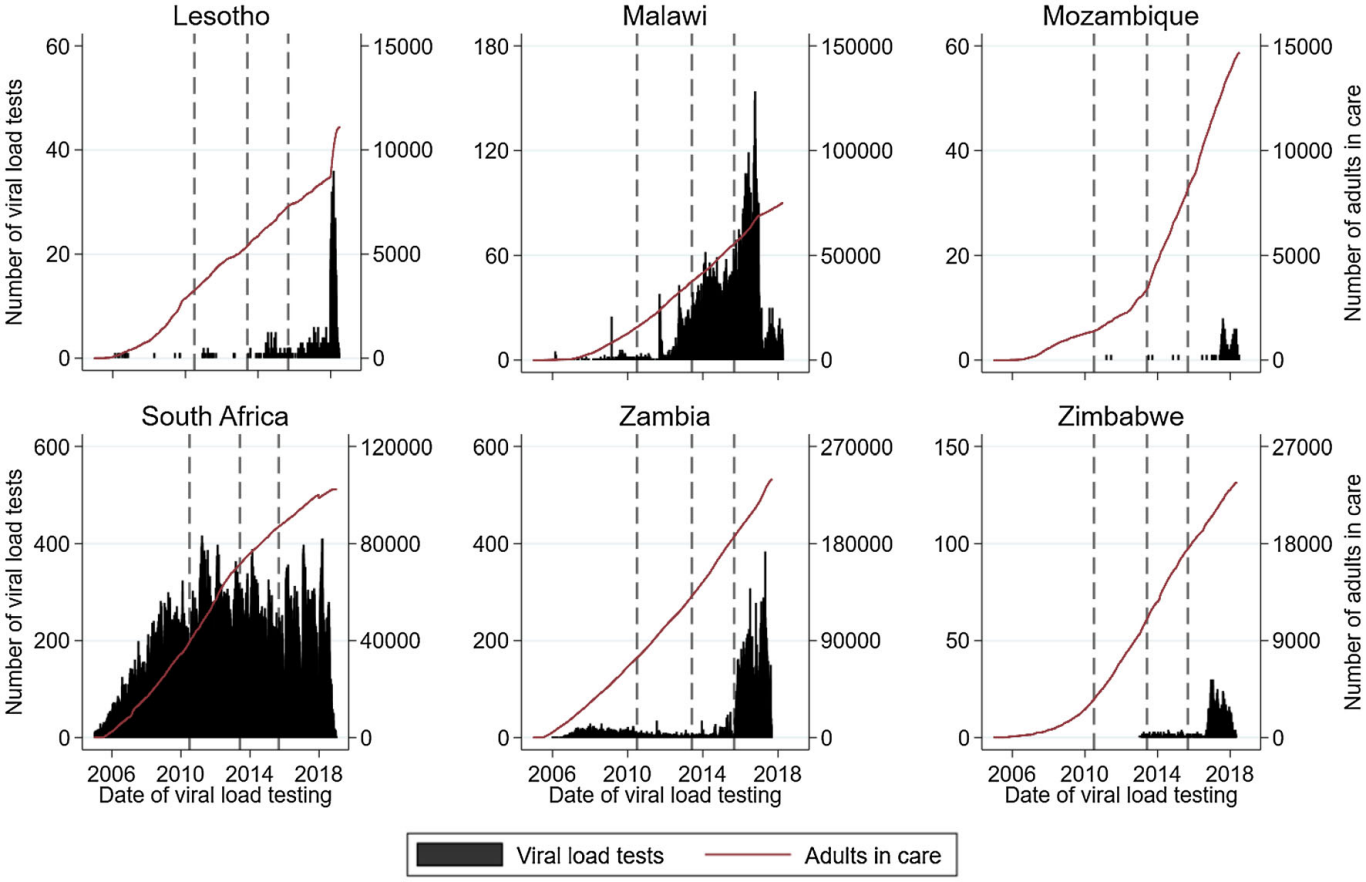
Frequency of viral load testing per day and cumulative number of adult patients in care by country. The vertical lines indicate the change in WHO guidelines. Scale of y‐axis differs across countries.

**Figure 3:**
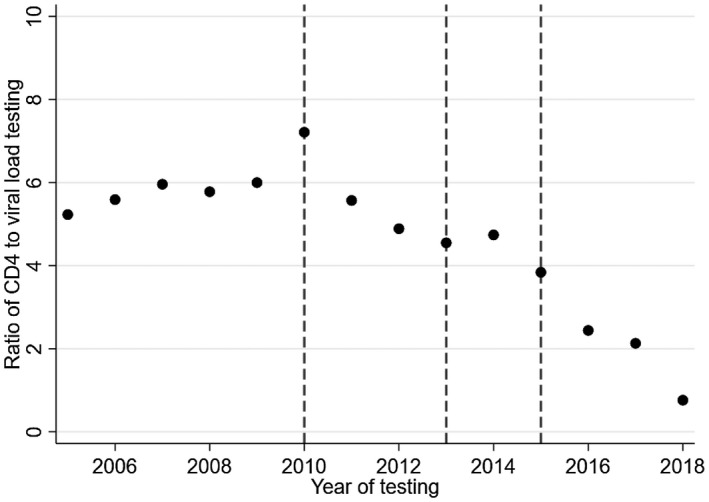
Trend of the ratio of CD4 cell count testing to viral load testing among adult patients by year of testing. The vertical lines indicate the change in WHO guidelines.

There was an increase in the frequency of CD4 testing alongside an increasing number of adults in care in earlier years (Figure 1). However, in all countries the frequency of CD4 testing plateaued or declined, typically after 2010, despite an increasing number of adults in care and under follow‐up in subsequent years; Malawi and South Africa had the strongest declines in testing. Figure 2 shows the frequency of VL testing. In South Africa, VL testing increased steeply in earlier years, but plateaued after 2011 despite an increasing number of adults in care. In Zambia, VL testing increased slightly in earlier years and then waned before increasing sharply in 2015. Malawi experienced a strong increase in VL testing after 2011. VL testing increased very recently in Lesotho, Mozambique and Zimbabwe. The ratio of CD4 to VL testing in the region decreased from a high of 7.2 in 2010 to a low of 0.76 in 2018 (Figure 3). The ratio also decreased in each of the six countries (Figure S1).

### 3.2 CD4 cell count testing at ART initiation

**Figure 4:**
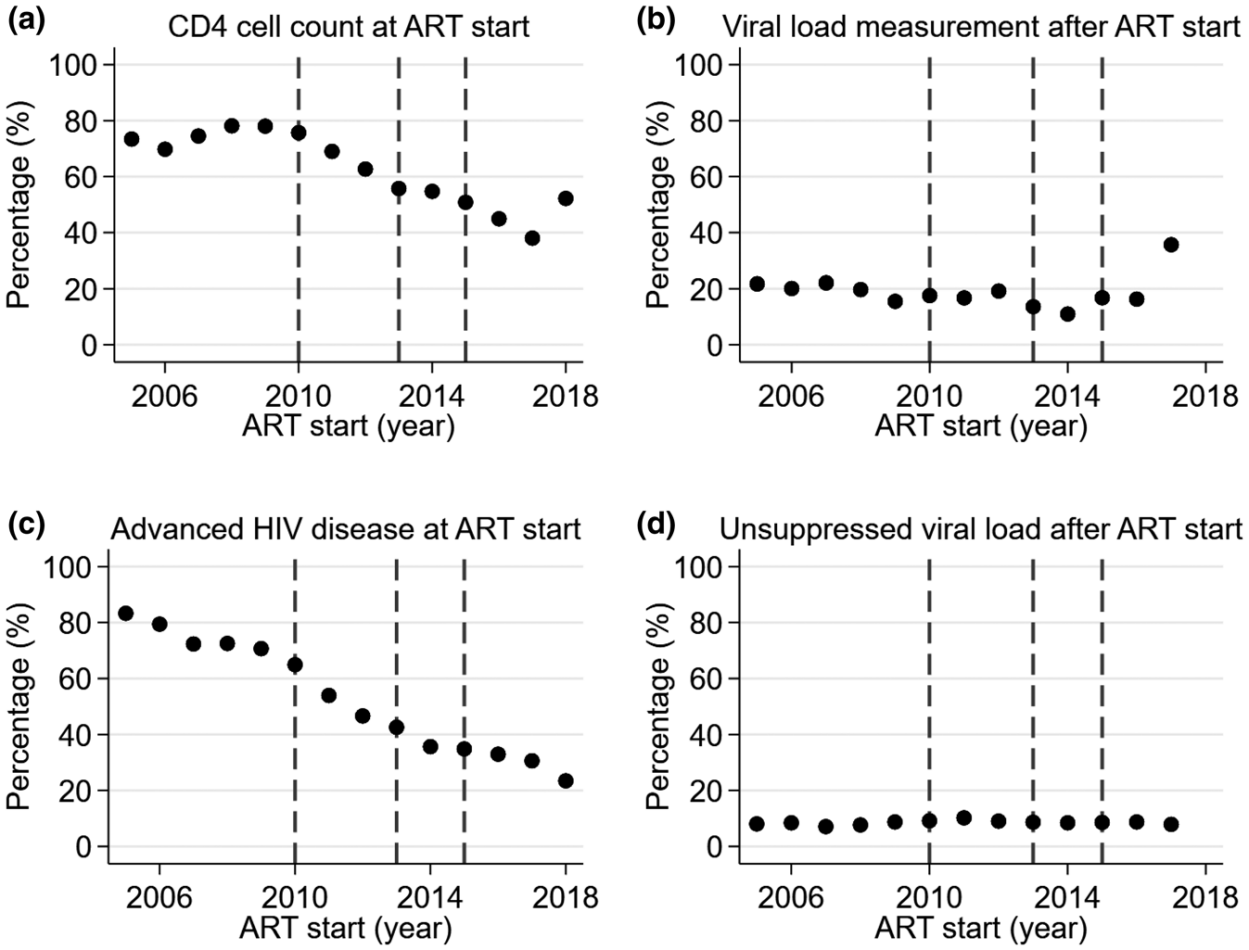
Trends of CD4 cell count testing at antiretroviral therapy (ART) start and viral load (VL) testing after ART start among adult patients by year of ART start. **(a)** The percentage of patients with a CD4 cell count at antiretroviral therapy (ART) start and, among those, **(c)** the percentage with advanced HIV disease; and **(b)** the percentage with a VL test three to nine months after ART start and, among those, **(d)** the percentage with unsuppressed VL by year of ART start. Advanced HIV disease defined as CD4 cell count <200 cells/mm^3^; unsuppressed VL defined as HIV‐1 RNA > 1000 copies/mL. The vertical lines indicate the change in WHO guidelines.

Overall, 61.4% of adults had a CD4 cell count at ART start, ranging from 31.0% in Malawi to 81.4% in South Africa (Table 2). The percentage of adults with a CD4 cell count at ART start decreased over the years from a peak of 78.1% in 2008 to a low of 38.0% in 2017 with an increase to 52.2% in 2018 (Figure 4). Among those with CD4 cell count testing at ART start, 53.6% had advanced HIV disease (Table 2). South Africa had the highest percentage of adults with advanced HIV disease (59.7%) and Mozambique the lowest (34.1%). The percentage of adults with advanced HIV disease steadily declined over time from a high of 83.3% in 2005 to a low of 23.5% in 2018 (Figure 4). Crude trends of the percentage of adults with a CD4 cell count test at ART start and, among those with CD4 testing, the percentage with advanced HIV disease by country are shown in Figure S2.

**Table 2:** CD4 cell count testing at antiretroviral therapy start (ART) and viral load (VL) testing after ART start among adult patients

Lesotho	Malawi	Mozambique	South Africa	Zambia	Zimbabwe	Total
CD4 cell count at ART start						
Total No. of patients						
12,441 (100%)	80,365 (100%)	16,178 (100%)	115,667 (100%)	251,816 (100%)	25,989 (100%)	502,456 (100%)
Patients with CD4 cell count testing at ART start						
9230 (74.2%)	24,925 (31.0%)	10,472 (64.7%)	94,167 (81.4%)	155,459 (61.7%)	14,199 (54.6%)	308,452 (61.4%)
Patients with advanced HIV disease among those with CD4 cell count testing at ART start						
3777 (40.9%)	13,004 (52.2%)	3570 (34.1%)	56,228 (59.7%)	81,450 (52.4%)	7249 (51.1%)	165,278 (53.6%)
VL testing after ART start						
Total No. of patients						
9386 (100%)	70,887 (100%)	14,698 (100%)	110,520 (100%)	228,699 (100%)	24,338 (100%)	458,528 (100%)
Patients with VL testing three to nine months after ART start						
41 (0.4%)	7574 (10.7%)	17 (0.1%)	67,409 (61.0%)	4222 (1.8%)	195 (0.8%)	79,458 (17.3%)
Patients with unsuppressed VL among those with VL testing three to nine months after ART start						
12 (29.3%)	646 (8.5%)	12 (70.6%)	5700 (8.5%)	442 (10.5%)	31 (15.9%)	6843 (8.6%)

Number of patients (%) are shown. Because of the shorter study period, the number of patients included in the analyses of VL testing after antiretroviral therapy (ART) start is smaller (n = 458,528) than the number included in the analysis of CD4 cell count testing at ART start (n = 502,456). Advanced HIV disease defined as CD4 cell count <200 cells/mm^3^; unsuppressed VL defined as HIV‐1 RNA > 1000 copies/mL.

The probability of having a CD4 cell count at ART start was 14% lower (odds ratio (OR) 0.86; 95% CI 0.86 to 0.86) and the odds of having advanced HIV disease was 20% lower each subsequent year (OR 0.80; 95% CI 0.80 to 0.81) (Table 3). Adults aged 25 to 49 and >49 years had a higher probability of CD4 cell count testing at ART start and a higher probability of advanced HIV disease than adults <25 years of age. Women had a lower probability of CD4 cell count testing at ART start (OR 0.86; 95% CI 0.85 to 0.87) than men and a lower probability of advanced HIV disease (OR 0.63; 95% CI 0.62 to 0.64).

**Table 3:** Probability of CD4 cell count testing at antiretroviral therapy (ART) start and viral load (VL) testing after ART start among adult patients

	CD4 cell count at ART start (N = 502,456)	Advanced HIV disease (N = 308,452)	VL after ART start (N = 458,528)	Unsuppressed VL (N = 79,458)
	OR (95% CI)	OR (95% CI)	OR (95% CI)	OR (95% CI)
Per calendar year	0.86 (0.86 to 0.86)	0.80 (0.80 to 0.81)	1.06 (1.05 to 1.06)	1.00 (0.99 to 1.01)
Age group in years				
<25	1	1	1	1
25 to 49	1.18 (1.16 to 1.21)	1.50 (1.47 to 1.54)	1.45 (1.40 to 1.50)	0.70 (0.65 to 0.76)
>49	1.20 (1.17 to 1.24)	1.48 (1.43 to 1.53)	1.34 (1.28 to 1.41)	0.52 (0.46 to 0.59)
Sex				
Male	1	1	1	1
Female	0.86 (0.85 to 0.87)	0.63 (0.62 to 0.64)	1.06 (1.04 to 1.09)	0.79 (0.75 to 0.83)

Results from the mixed‐effects logistic regression model showing the probability of having a CD4 cell count at antiretroviral therapy (ART) start, a VL test three to nine months after ART start and, among those with testing, the probability of having advanced HIV disease and unsuppressed VL by calendar year, age and sex. CD4 cell count analyses based on 502,456 adults of whom 308,452 (61.4%) had CD4 cell count testing at ART start; VL analyses based on 458,528 adults of whom 79,458 (17.3%) had VL test testing after ART start. Advanced HIV disease defined as CD4 cell count <200 cells/mm^3^; unsuppressed VL defined as HIV‐1 RNA > 1000 copies/mL.

Expanding the CD4 cell count testing time window from three to six months before ART initiation increased the percentage of adults with a CD4 cell count at ART start, with the difference decreasing over time (Figure S3).

### 3.3 VL testing after ART initiation

Overall, 17.3% of adults had a VL test three to nine months after ART start, among whom 8.6% were unsuppressed (Table 2). South Africa had the highest percentage of adults with VL testing after ART start (61.0%), followed by Malawi (10.7%); VL testing was below 2% in all other countries. Among adults with VL testing, the percentage with unsuppressed VL was lowest in South Africa and Malawi (8.5% and 8.5% respectively) and highest in Mozambique (70.6%). Between 2005 and 2016, the percentage with VL testing fluctuated between 10% and 23%; this increased to 35.7% in 2017 (Figure 4). For all years, the percentage with unsuppressed VL among those with VL testing ranged between 7% and 11%. Crude trends of the percentage of adults with a VL test after ART start and, among those with VL testing, the percentage with unsuppressed VL by country are shown in Figure S2.

The probability of having a VL test three to nine months after ART start increased modestly over time (OR 1.06; 95% CI 1.05 to 1.06), but there was no evidence of a decrease in unsuppressed VL (OR 1.00; 95% CI 0.99 to 1.01) (Table 3). The probability of having a VL measurement after ART start was higher for adults aged 25 to 49 years and >49 years than for adults aged <25 years. However, among those with VL testing, the probability of having an unsuppressed VL was lower for adults aged 25 to 49 and >49 years than for adults aged <25 years. Women had a slightly higher probability of having a VL test after ART start (OR 1.06; 95% CI 1.04 to 1.09) and a lower probability of unsuppressed VL (OR 0.79; 95% CI 0.75 to 0.83) than men.

## 4 DISCUSSION

This large study of CD4 and VL testing in ART programmes from six Southern African countries showed the percentage of adults with a CD4 cell count at the start of ART declined from a peak of about 78% in 2008 to below 40% in 2017. Among those with a CD4 count, the percentage starting with advanced HIV disease (<200 cells/mm^3^) also declined, but almost a quarter of patients still started ART with advanced HIV disease in 2018. VL testing increased in the earlier years in South Africa and then plateaued. In the other countries testing increased in more recent years, but remained modest considering the large number of patients in care. In 2017, overall only about a third of patients had a VL measurement after the start of ART.

The trends in CD4 testing observed in this study likely reflect changes in ART guidelines. In earlier years, when ART eligibility was governed by relatively low CD4 thresholds (<200 cells/mm^3^ up to 2010 [25], ≤350 up to 2013 [26, 27]), repeated CD4 counts were needed to identify when patients became eligible for ART. As eligibility expanded to higher CD4 thresholds and, in September 2015, “Treat all” guidance was introduced recommending all individuals be treated as soon as possible after diagnosis of HIV infection, less repeat CD4 testing was required [28]. Of note, the peak in the ratio of CD4 to VL testing in 2010 coincides with the release of guidelines recommending ART during pregnancy and breastfeeding to reduce vertical transmission under Option B [29]. In the earlier years, WHO and national guidelines recommended routine CD4 cell count monitoring for patients on ART [27, 30]. However, in 2012 the Southern African HIV Clinicians Society released new guidelines recommending VL monitoring rather than routine CD4 cell count monitoring for patients on ART with a CD4 cell count above 200 cells/mm^3^ [31]. The following year, the WHO and the South African Department of Health released updated guidelines no longer recommending routine CD4 cell count monitoring for patients on ART where VL monitoring was assured [12, 32].

The decline in CD4 cell count testing at the start of ART observed in these six countries and in similar studies is of concern [33, 34]. Although CD4 cell count thresholds for starting ART and CD4 cell count monitoring on ART are no longer recommended in the “Treat all” era, WHO still recommends baseline CD4 cell count testing for all patients entering care and before ART initiation to identify patients with advanced HIV disease [5, 7]. CD4 cell count testing remains important because clinical staging has limited diagnostic accuracy for identifying patients with low CD4 cell count [35]. WHO recommends a package of interventions among patients with advanced HIV disease, including screening, treatment and/or prophylaxis for major opportunistic infections, rapid ART initiation and intensified adherence support [8]. Scaling up access to this package of interventions to reduce mortality and morbidity is also being supported by major donors such as the International Drug Purchase Facility (UNITAID) [36]. The decrease in the proportion of patients with a CD4 cell count at the start of ART indicates that many patients with advanced HIV disease may be missed. The percentage with a CD4 count increased in 2018 to 52.2%. Future analyses of the IeDEA cohorts will examine whether or not this reversal of the trend is sustained.

PEPFAR, which provided substantial support to all countries included in this study, recently shifted their policy away from routine baseline CD4 cell count testing to prioritize access to VL monitoring [16, 17]. PEPFAR continues to support limited CD4 testing in countries where more than 10% of patients start ART with advanced HIV disease [16, 17]. We found that almost a quarter of patients with a CD4 count started ART with advanced HIV disease in 2018, but many started without a CD4 cell count. A recent multiregional analysis of IeDEA data used multiple imputation to adjust for missing CD4 cell counts and found that although median CD4 cell counts at ART start increased substantially from 2002 to 2015, the proportion with advanced disease (CD4 cell count <200 cells/mm^3^) was around 40% in low‐income and middle‐income countries in 2015 [37]. It is likely that the proportion of patients with advanced HIV disease at the time of initiating ART is above the PEPFAR threshold of 10%, indicating the need for continued baseline CD4 cell count testing.

VL testing is the preferred method for monitoring patients on ART, as treatment failure is identified earlier and more accurately than CD4 cell count monitoring [5, 38]. Despite the recommendation to switch from CD4 to VL monitoring, a substantial increase in VL testing parallel to the decrease in CD4 count testing was evident only in South Africa and Malawi. In the other four countries included in this study, Lesotho, Mozambique, Zambia and Zimbabwe, the scale‐up of VL testing started only very recently. Similar trends have been observed in other studies of countries in sub‐Saharan Africa [33, 39, 40]. The higher rate of unsuppressed VL in these latter countries further indicates they are still transitioning from targeted to routine VL testing.

Two major strengths of this study are the timeliness of data and the large number of patients: we included data reported up to 2018 on more than 500,000 people living with HIV in six countries in Southern Africa. Our study also has several limitations. The ART programmes and clinics participating in IeDEA Southern Africa may not represent all treatment facilities located in the region; therefore, the findings in this study may not be applicable to the country or region as a whole. The large heterogeneity observed in laboratory testing frequencies among countries in the region further limits the interpretation of our regional conclusions. Our study did not account for differences in level of care or access to laboratory testing that may vary across and within countries and be a result of different facility type and location. Our study also did not take into account or attempt to correct for missing laboratory data. Our study focused on patients who started ART and did not consider patients lost to follow‐up before or after ART initiation, patients who silently transferred to another ART programme, or unreported mortality. Lastly, we were unable to ascertain trends in CD4 cell count testing among patients with suspected treatment failure, which is recommended by the WHO and many national guidelines. Although this study analysed recently collected data, the findings will not reflect the most recent policy changes.

## 5 Conclusions

This large multi‐cohort study of people living with HIV in Southern Africa showed that CD4 cell count testing declined over time, including testing at the start of ART, despite the fact that many patients still initiate ART with advanced HIV disease. Although guidelines and national policies support the scale‐up of VL testing, the coverage of VL testing was low in most countries. Without CD4 cell count testing and expanded VL testing many patients with advanced HIV disease and treatment failure may go undetected, threatening the effectiveness of ART in Southern Africa.

## COMPETING INTERESTS

The authors declare that they have no competing interests.

## AUTHORS’ CONTRIBUTIONS

EZ, ME, NF, FC, MAD, JVD, JE and MPF conceptualized the study, while EZ, CHDO, NM, MAD, JE, JVD, SB, SP, FT, NS, JM, GF, HP, RW, MPF and ME contributed to data collection. EZ, FC and ME analysed the data and interpreted the results. EZ wrote the first draft of the manuscript, which was revised by all authors. All authors have reviewed and approved the final version of the manuscript.

## Supporting information


**Table S1.** Characteristics of adult patients (aged ≥15 years) at antiretroviral therapy (ART) initiation by country for the sub‐analysis of viral load testing after ART start.

**Figure S1.** Trends of the ratio of CD4 cell count testing to viral load testing among adult patients (aged ≥15 years) by year of testing and country. The vertical lines indicate the change in WHO guidelines. N/A: no CD4 cell count or viral load testing data available for patients in that year.

**Figure S2.** Trends of CD4 cell count testing at antiretroviral therapy (ART) start and viral load (VL) testing after ART start by country. The percentage of adult patients (aged ≥15 years) with a CD4 cell count at initiation of antiretroviral therapy (ART) and, among those, the percentage with advanced HIV disease; and the percentage with a VL test three to nine months after ART start and, among those, the percentage with unsuppressed VL by year of ART start. The vertical lines indicate the change in WHO guidelines. N/A: no CD4 cell count or VL testing data available for patients in that year. Advanced HIV disease defined as CD4 < 200 cells/mm^3^; unsuppressed viral load defined as measurement HIV‐1 RNA > 1000 copies/mL.

**Figure S3.** Trends of CD4 cell count testing three months versus six months before antiretroviral therapy start among adult patients. The percentage of adult patients (aged ≥15 years) with a CD4 cell count up to three months before antiretroviral therapy (ART) start and up to six months before ART start and, among those with testing, the percentage with advanced HIV disease by year of ART start. The vertical lines indicate the change in WHO guidelines. Advanced HIV disease defined as CD4 < 200 cells/mm^3^.
