# Supplementary material for: Trends in CD4 and viral load testing 2005 to 2018: multi‐cohort study of people living with HIV in Southern Africa
Source: J Int AIDS Soc. 2020 Jul 8;23(7):e25546. doi: 10.1002/jia2.25546 (PMC7343336; doi:10.1002/jia2.25546)
Supplement: Supplementary file 1 — Table S1. Characteristics of adult patients (aged ≥15 years) at antiretroviral therapy (ART) initiation by country for the sub‐analysis of viral load testing after ART start. [file JIA2-23-e25546-s004.pdf]

Table S1: Characteristics of adult patients (aged ≥15 years) at antiretroviral therapy (ART) initiation by country for the sub-analysis of viral load testing after ART start.

	Lesotho	Malawi	Mozambique	South Africa	Zambia	Zimbabwe	Total
**Total No. of patients**	9,386 (100%)	70,887 (100%)	14,698 (100%)	110,520 (100%)	228,699 (100%)	24,338 (100%)	458,528 (100%)
**Female**	6,202 (66.1%)	43,448 (61.3%)	10,001 (68.0%)	74,196 (67.1%)	142,222 (62.2%)	16,638 (68.4%)	292,707 (63.8%)
**Age group** in years							
<25	852 (9.1%)	9,177 (13.0%)	3,507 (23.9%)	11,935 (10.8%)	27,144 (11.9%)	2,624 (10.8%)	55,239 (12.0%)
25-49	6,726 (71.6%)	54,751 (77.2%)	9,910 (67.4%)	88,674 (80.2%)	182,470 (79.8%)	17,784 (73.1%)	360,315 (78.6%)
>49	1,808 (19.3%)	6,959 (9.8%)	1,281 (8.7%)	9,911 (9.0%)	19,085 (8.3%)	3,930 (16.1%)	42,974 (9.4%)
**Median (IQR) follow-up** in months	58.8 (30.5-87.3)	55.8 (30.1-82.0)	43.9 (25.4-63.6)	77.6 (43.2-109.7)	67.3 (36.6-102.4)	60.6 (37.8-85.5)	65.1 (36.5-98.5)

Number of patients (%) are shown unless otherwise indicated.
