# Supplementary material for: Trends in CD4 and viral load testing 2005 to 2018: multi‐cohort study of people living with HIV in Southern Africa
Source: J Int AIDS Soc. 2020 Jul 8;23(7):e25546. doi: 10.1002/jia2.25546 (PMC7343336; doi:10.1002/jia2.25546)
Supplement: Supplementary file 2 — Figure S1. Trends of the ratio of CD4 cell count testing to viral load testing among adult patients (aged ≥15 years) by year of testing and country. The vertical lines indicate the change in WHO guidelines. N/A: no CD4 cell count or viral load testing data available for patients in that year. [file JIA2-23-e25546-s001.pdf]

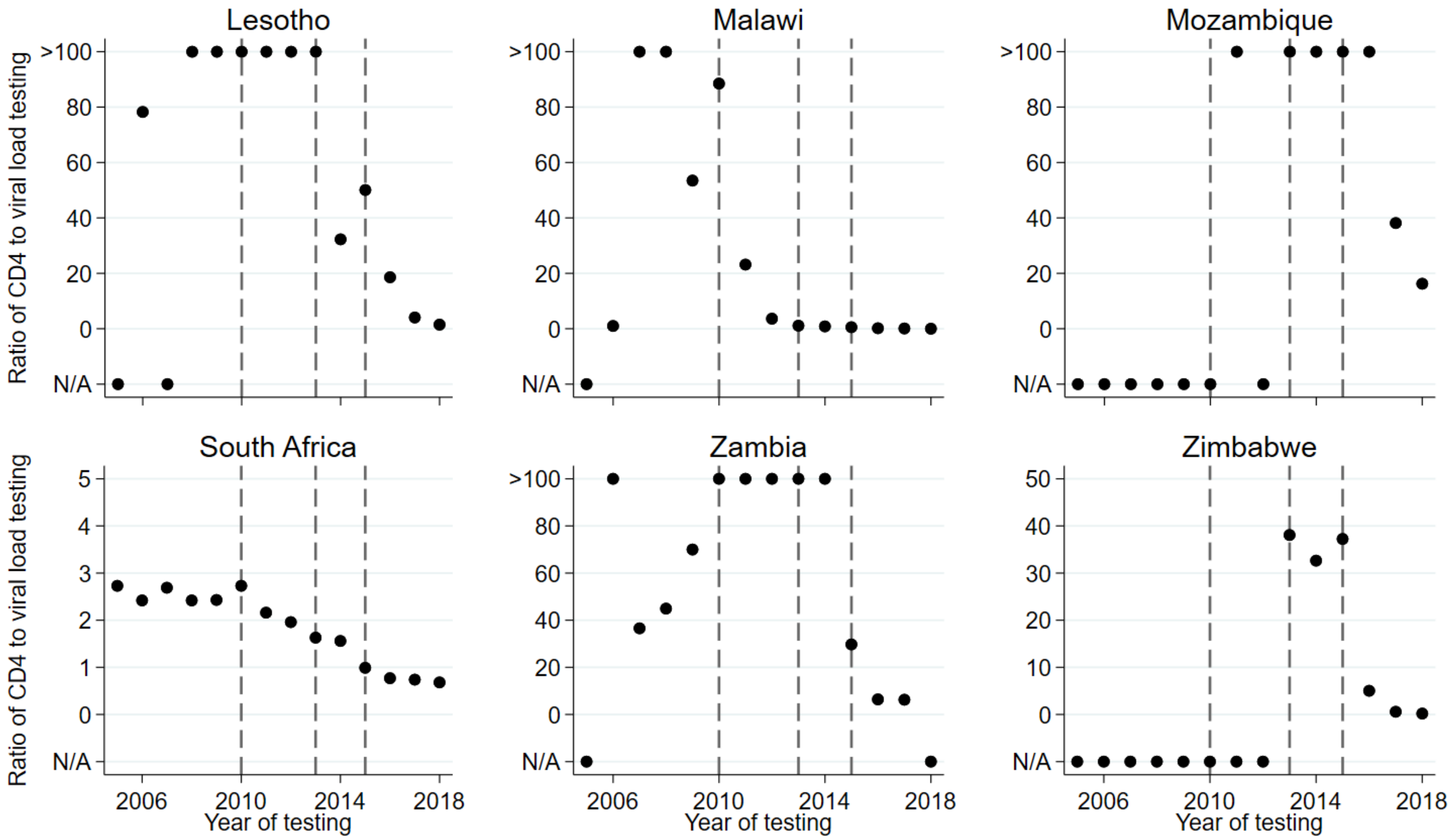

Figure S1: Trends of the ratio of CD4 cell count testing to viral load testing among adult patients (aged ≥15 years) by year of testing and country.

The vertical lines indicate the change in WHO guidelines. N/A: no CD4 cell count or viral load testing data available for patients in that year.
