# Supplementary material for: Trends in CD4 and viral load testing 2005 to 2018: multi‐cohort study of people living with HIV in Southern Africa
Source: J Int AIDS Soc. 2020 Jul 8;23(7):e25546. doi: 10.1002/jia2.25546 (PMC7343336; doi:10.1002/jia2.25546)
Supplement: Supplementary file 3 — Figure S2. Trends of CD4 cell count testing at antiretroviral therapy (ART) start and viral load (VL) testing after ART start by country. The percentage of adult patients (aged ≥15 years) with a CD4 cell count at initiation of antiretroviral therapy (ART) and, among those, the percentage with advanced HIV disease; and the percentage with a VL test three to nine months after ART start and, among those, the percentage with unsuppressed VL by year of ART start. The vertical lines indicate the change in WHO guidelines. N/A: no CD4 cell count or VL testing data available for patients in that year. Advanced HIV disease defined as CD4 < 200 cells/mm3; unsuppressed viral load defined as measurement HIV‐1 RNA > 1000 copies/mL. [file JIA2-23-e25546-s002.pdf]

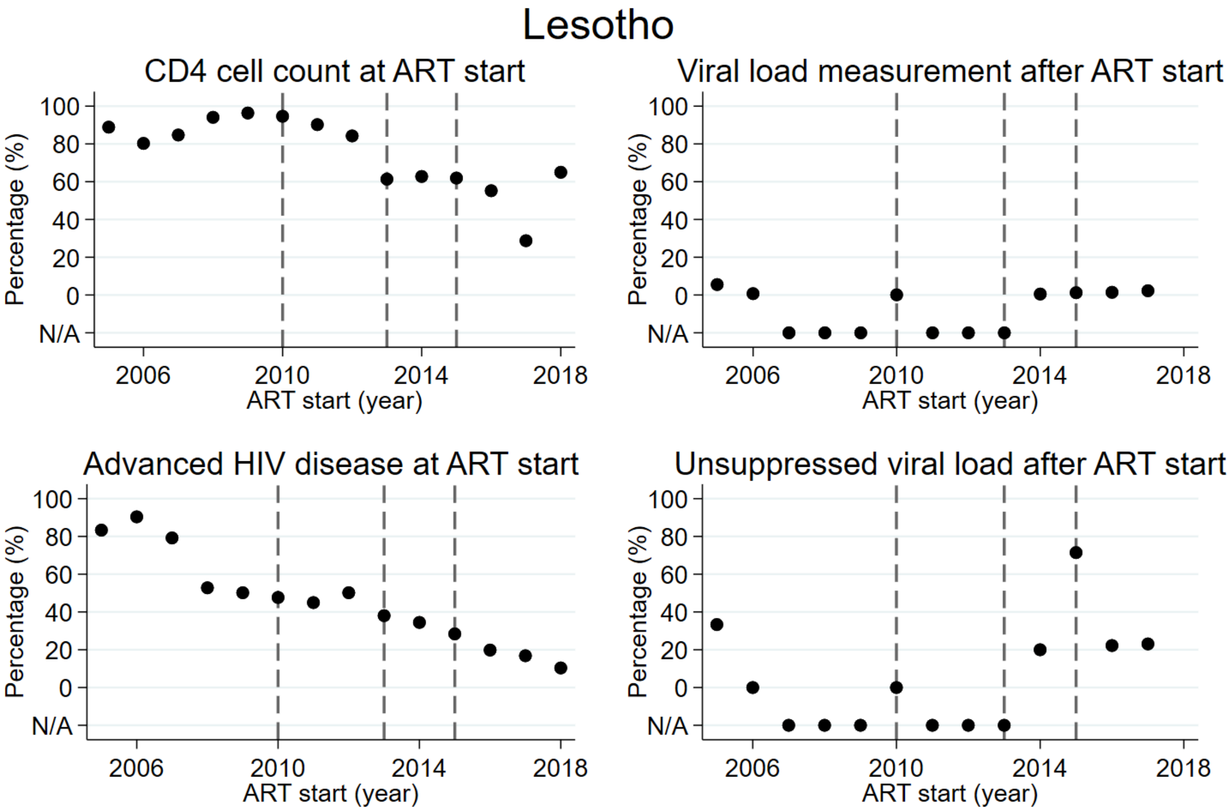
Lesotho
CD4 cell count at ART start
Viral load measurement after ART start
Advanced HIV disease at ART start
Unsuppressed viral load after ART start
Percentage (%)
100
80
60
40
20
0
N/A
2006
2010
2014
2018
ART start (year)

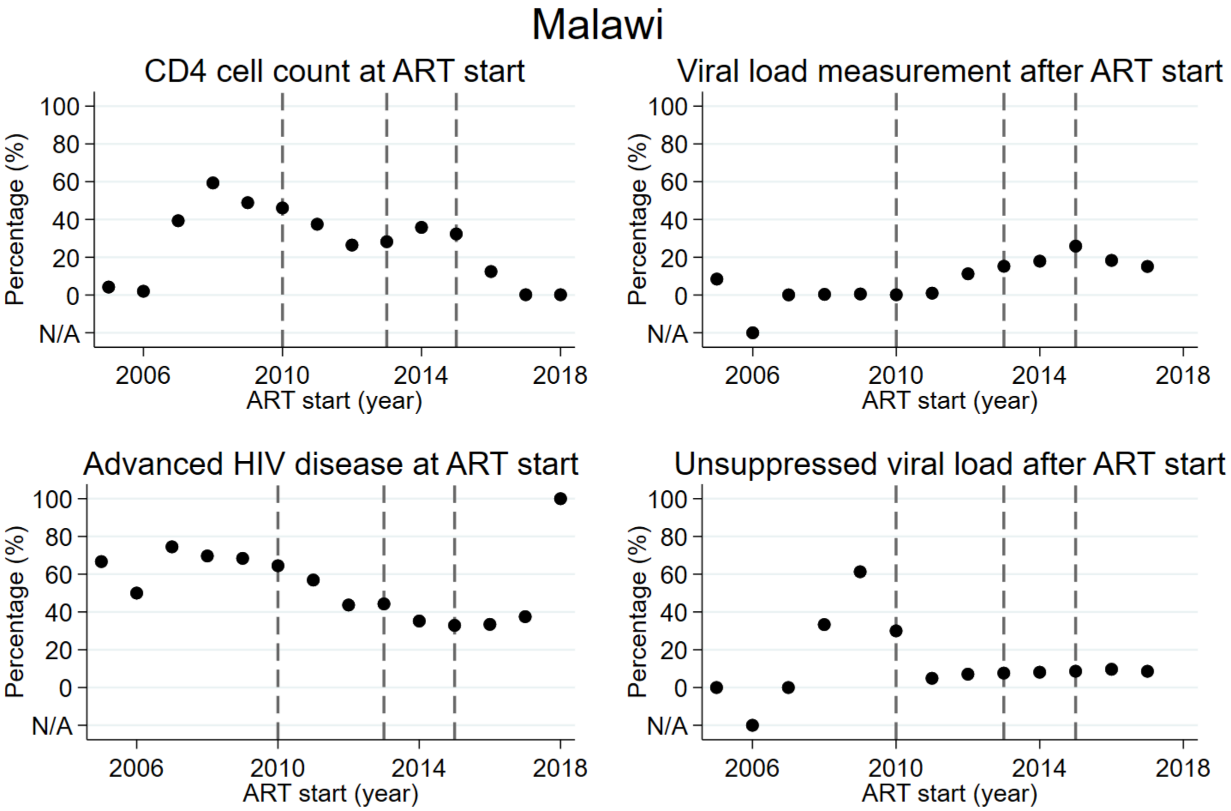
Malawi
CD4 cell count at ART start
Viral load measurement after ART start
Advanced HIV disease at ART start
Unsuppressed viral load after ART start
Percentage (%)
100
80
60
40
20
0
N/A
2006
2010
2014
2018
ART start (year)

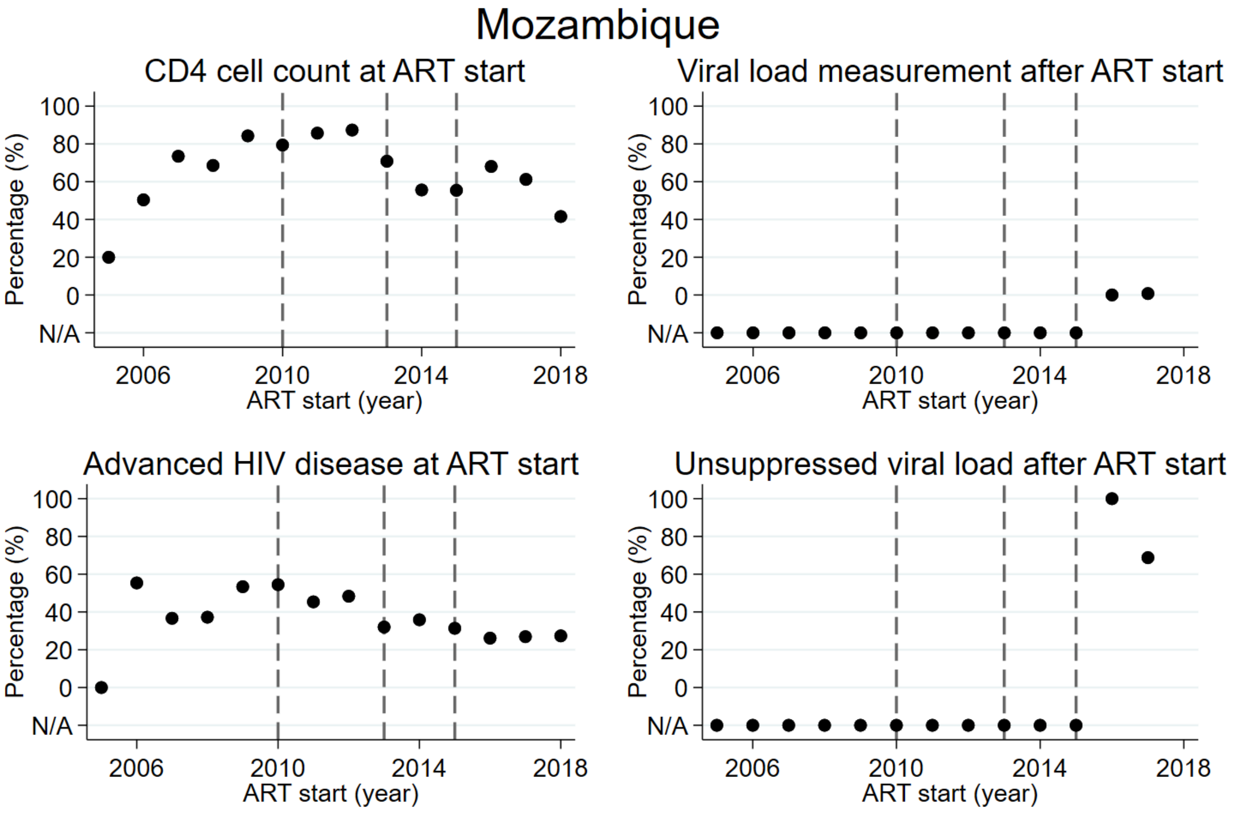
Mozambique
CD4 cell count at ART start
Viral load measurement after ART start
Advanced HIV disease at ART start
Unsuppressed viral load after ART start
Percentage (%)
100
80
60
40
20
0
N/A
2006
2010
2014
2018
ART start (year)

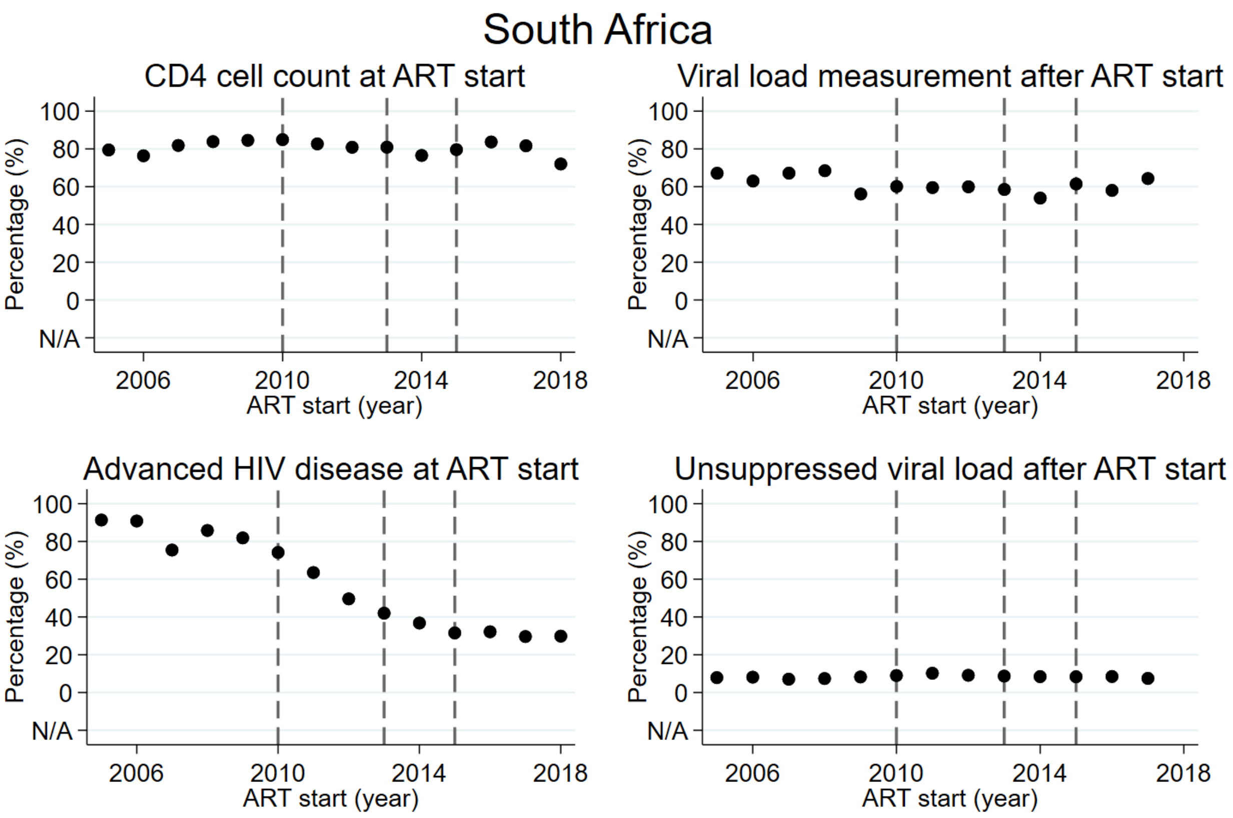
South Africa
CD4 cell count at ART start
Viral load measurement after ART start
Advanced HIV disease at ART start
Unsuppressed viral load after ART start
Percentage (%)
100
80
60
40
20
0
N/A
2006
2010
2014
2018
ART start (year)

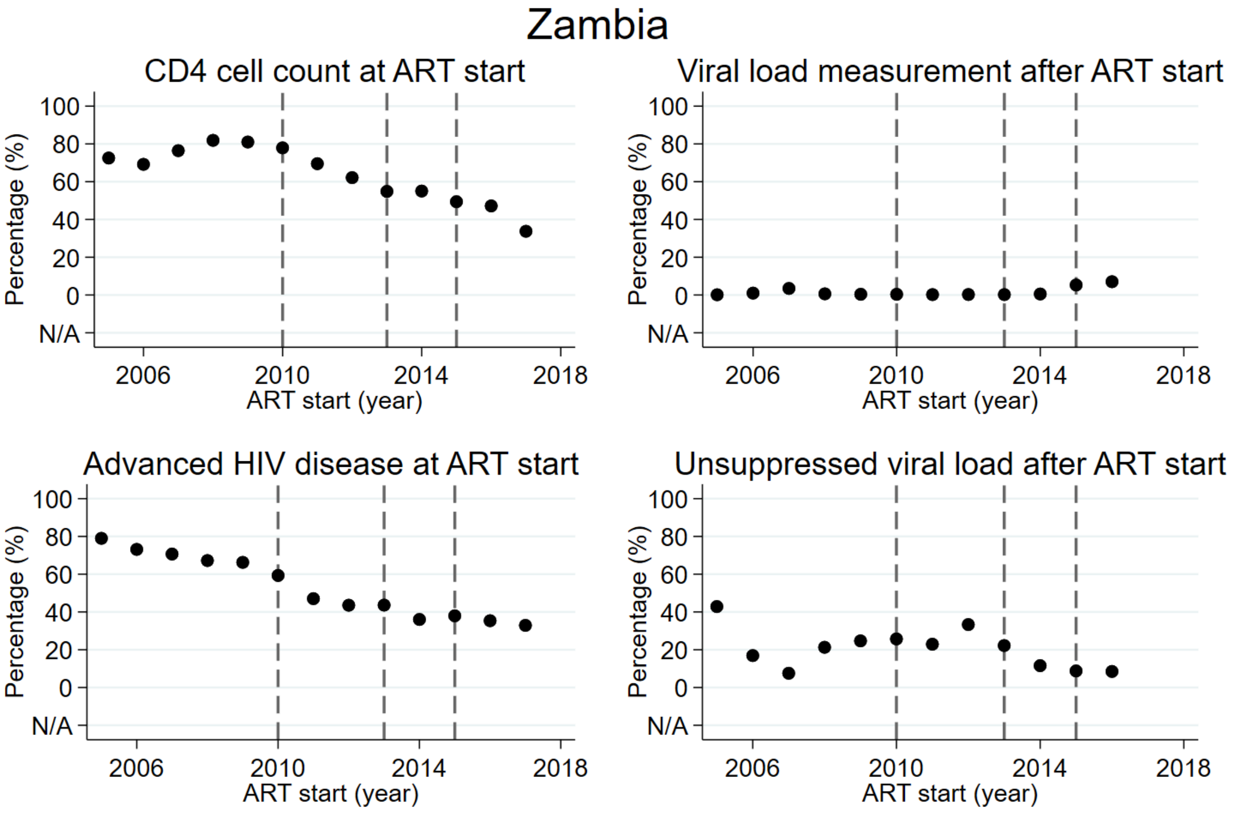
Zambia
CD4 cell count at ART start
Viral load measurement after ART start
Advanced HIV disease at ART start
Unsuppressed viral load after ART start
Percentage (%)
100
80
60
40
20
0
N/A
2006
2010
2014
2018
ART start (year)

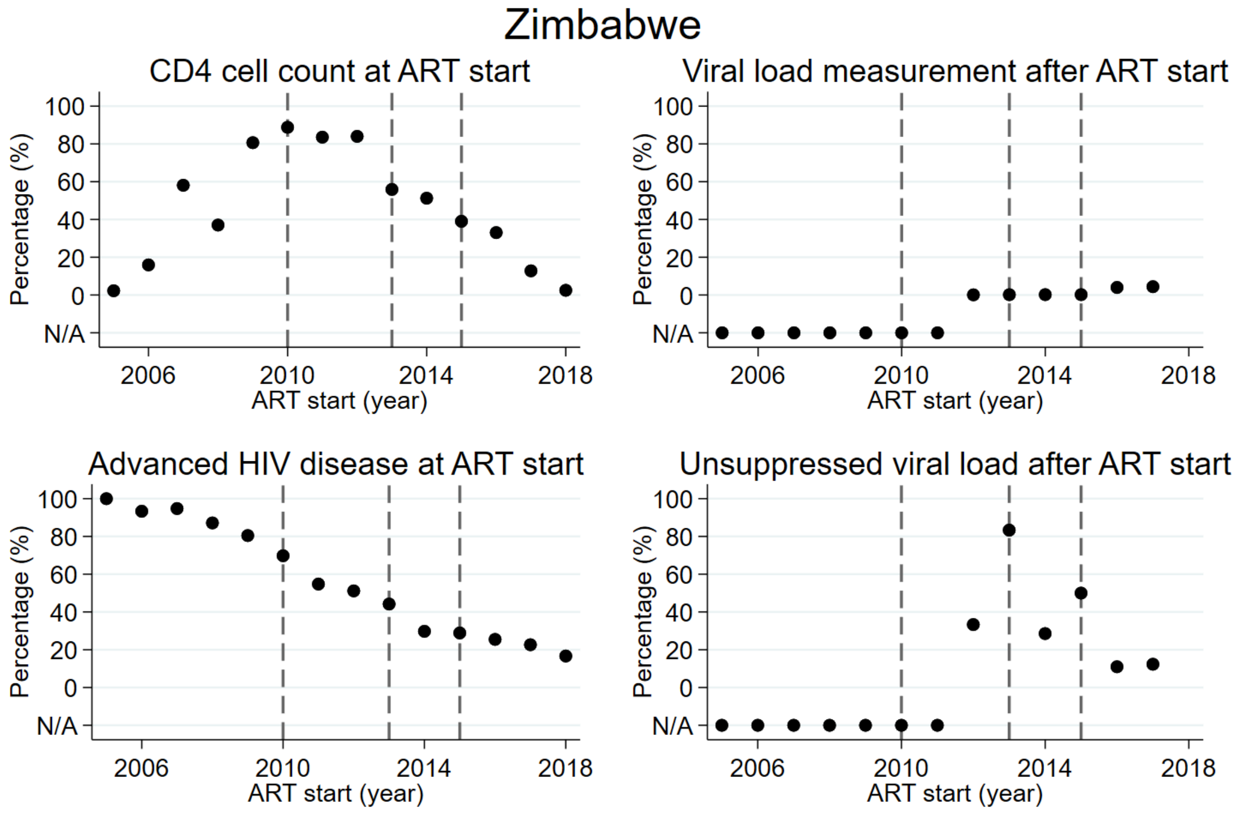

Figure S2: Trends of CD4 cell count testing at antiretroviral therapy (ART) start and viral load testing after ART start by country.

The percentage of adult patients (aged ≥15 years) with a CD4 cell count at initiation of antiretroviral therapy (ART) and, among those, the percentage with advanced HIV disease; and the percentage with a viral load test 3-9 months after ART start and, among those, the percentage with unsuppressed viral load by year of ART start. The vertical lines indicate the change in WHO guidelines. N/A: no CD4 cell count or viral load testing data available for patients in that year. Advanced HIV disease defined as CD4 <200 cells/mm^3; unsuppressed viral load defined as measurement HIV-1 RNA >1000 copies/ml.
