# Supplementary material for: Trends in CD4 and viral load testing 2005 to 2018: multi‐cohort study of people living with HIV in Southern Africa
Source: J Int AIDS Soc. 2020 Jul 8;23(7):e25546. doi: 10.1002/jia2.25546 (PMC7343336; doi:10.1002/jia2.25546)
Supplement: Supplementary file 4 — Figure S3. Trends of CD4 cell count testing three months versus six months before antiretroviral therapy start among adult patients. The percentage of adult patients (aged ≥15 years) with a CD4 cell count up to three months before antiretroviral therapy (ART) start and up to six months before ART start and, among those with testing, the percentage with advanced HIV disease by year of ART start. The vertical lines indicate the change in WHO guidelines. Advanced HIV disease defined as CD4 < 200 cells/mm3. [file JIA2-23-e25546-s003.pdf]

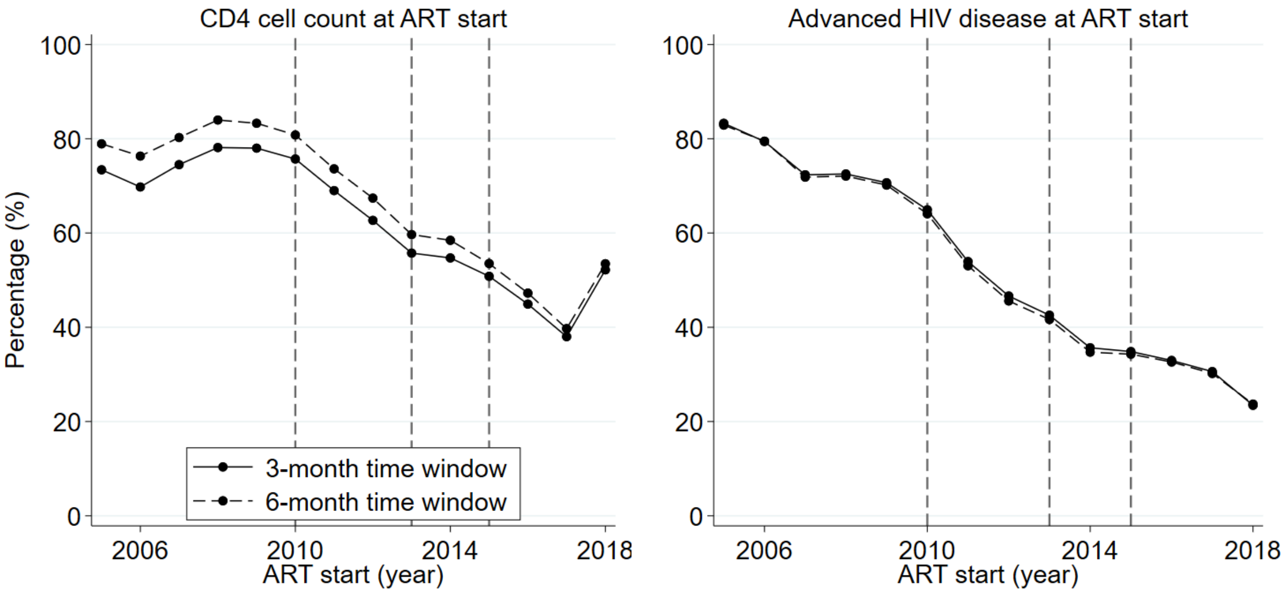

Figure S3: Trends of CD4 cell count testing three months vs six months before antiretroviral therapy start among adult patients.

The percentage of adults patients (aged ≥15 years) with a CD4 cell count up to three months before antiretroviral therapy (ART) start and up to six months before ART start and, among those with testing, the percentage with advanced HIV disease by year of ART start. The vertical lines indicate the change in WHO guidelines. Advanced HIV disease defined as CD4 <200 cells/mm^3.
